# Fentanyl Promotes Breast Cancer Cell Stemness and Epithelial-Mesenchymal Transition by Upregulating α1, 6-Fucosylation via Wnt/β-Catenin Signaling Pathway

**DOI:** 10.3389/fphys.2017.00510

**Published:** 2017-07-26

**Authors:** Hong-Fang Yang, Ming Yu, Hui-Dan Jin, Jia-Qi Yao, Zhi-Li Lu, Iddrisu B. Yabasin, Qiu Yan, Qing-Ping Wen

**Affiliations:** ^1^Department of Anesthesiology, First Affiliated Hospital of Dalian Medical University Dalian, China; ^2^Liaoning Provincial Core Lab of Glycobiology and Glycoengineering, Department of Biochemistry and Molecular Biology, Dalian Medical University Dalian, China; ^3^Department of Anesthesiology, Affiliated Xinhua Hospital of Dalian University Dalian, China

**Keywords:** fentanyl, fucosyltransferase VIII, α1, 6-fucosylation, stemness, epithelial-mesenchymal transition, Wnt/β-catenin signaling pathway, breast cancer

## Abstract

Cancer pain is a common and severe complication of human breast cancer, and relieving pain is fundamental strategy in the treatment. Fentanyl, as an opioid analgesic, is widely used in breast cancer patients. However, little is known about its effects on stemness and epithelial-mesenchymal transition (EMT) of breast cancer cells. Aberrant protein glycosylation is involved in cancer malignancy. The α1, 6-fucosylation is an important type of glycosylation, and the elevated α1, 6-fucosylation catalyzed by fucosyltransferase VIII (FUT8) is found in many tumors. However, whether 1, 6-fucosylation is involved in regulating stemness and EMT, and stimulated by fentanyl is not clear. In this study, we found that fentanyl induced stemness and EMT in MCF-7 and MDA-MB-231 breast cancer cells by analysis of sphere formation, expression of stemness markers (Sox2, Oct4) and EMT markers (N-cadherin, E-cadherin and Vimentin). Results also showed that fentanyl upregulated FUT8 gene and protein expression by qPCR, Western blot and immunofluorescent staining, as well as α1, 6-fucosylation level by Lectin blot and Lectin fluorescent staining. Furthermore, decreased or blocked α1, 6-fucosylation by FUT8 siRNA transfection or LCA Lectin blockage reduced stemness and EMT. Additionally, fentanyl activated the key molecules and target genes in Wnt/β-catenin signaling pathway. LGK-974 (an inhibitor of Wnt ligands) suppressed fentanyl-mediated upregulation of α1, 6-fucosylation, stemness and EMT. The results of tumor xenograft demonstrated that fentanyl enhanced tumor growth, α1, 6-fucosylation, stemness and EMT. Taken together, our study reveals that fentanyl upregulated FUT8 expression, which increased α1, 6-fucosylation level through activation of Wnt/β-catenin signaling pathway, thereby, induce stemness and EMT of breast cancer cells. This study suggest a potential side effect of fentanyl in the treatment of cancer, which may guide the safety of fentanyl in the clinical application.

## Introduction

Breast cancer is the most common malignant tumor among women, and its incidence has been increasing annually worldwide (Siegel et al., 2013). Although the advances in oncological therapy have improved the survival rates of breast cancer patients, a large number of patients still suffer from cancer-related pains caused by surgical operation, tumor progression and metastasis, etc. (Miller et al., 2016). This leads to the patients' constant demand for the analgesic therapy during cancer treatment. Opioid analgesics, such as morphine and fentanyl, at the third step are extensively applied to relief pain according to the WHO step ladder for cancer pain management (Mercadante, 2010). Fentanyl is a completely synthetic opioid analgesic with the analgesic effect 60–100 times stronger than that of morphine (Trescot et al., 2008). It significantly minimized the surgical-induced upsurge of cardiovascular parameters (blood pressure, heart rate and myocardial oxygen consumption). As a result of the aforementioned advantages, fentanyl has become a commonly used analgesic for the management of pain during surgical resection of tumors and in the terminal cancer patients (Stanley, 2014). However, some previous studies showed that opioid drugs promoted tumor growth and metastasis (Afsharimani et al., 2011), angiogenesis (Gupta et al., 2002), as well as drug resistance (Niu et al., 2015). Although fentanyl is proven to be potent and reliable against acute and chronic pain, its influence on the stemness and EMT of human cancer cells is yet to be elucidated.

Cancer stem-like cells (CSCs) are very small subpopulation of cells in tumor population that display stemness including self-renewal, unlimited proliferation and differentiation (Czerwinska and Kaminska, 2015). The existence of CSCs has been identified in a variety of tumors, such as hematopoietic, brain, breast, colon malignancies etc. (Mani et al., 2008). Stemness is thought to be associated with tumorigenesis, progression, drug resistance and recurrence (Hermann et al., 2007; Mao et al., 2014). Once the cancer cells acquire stemness features, they generally express some specific stemness markers, such as transcription factors (Nanog, Sox2 and Oct4), which are essential for the maintenance of self-renewal and pluripotency (Pan and Thomson, 2007; Stadtfeld and Hochedlinger, 2010). Our previous results demonstrated that morphine could induce stemness of breast cancer cells (Niu et al., 2015). However, little is known about the effect of fentanyl on induction of stemness.

Epithelial-mesenchymal transition (EMT) is a complex cellular program by which epithelial cells lose their epithelial features to transform into mesenchymal cell phenotype (Boyer and Thiery, 1993). During this process, the expression of epithelial cell marker E-cadherin is decreased, which reduces the number of epithelial cell junctions; while the expression of mesenchymal cell markers, such as N-cadherin and Vimentin, are increased for the cells to gain ability of migration and invasion (Kalluri and Neilson, 2003). It is well known that EMT is associated with not only embryonic development and wound healing, but also tumor vascularization, invasiveness and metastasis, as well as the stemness (Ansieau, 2013). EMT can be caused by many factors, such as adverse conditions (Wang X. et al., 2016), extracellular stimuli (Juang et al., 2016), inflammatory cytokines (West et al., 2014) and small molecule drugs (Tian et al., 2016). However, whether fentanyl can alter the property of EMT in breast cancer cells remains largely unknown.

Protein glycosylation is one of the common post-translational modification steps. Glycosylation is involved in a variety of physiological and pathological processes (Stowell et al., 2015). It is considered that dysregulated glycosylation plays an important role in tumor malignancy, and acts as specific biomarker for the diagnosis of cancers (Fuster and Esko, 2005). Protein glycosylation is mainly divided into N- and O- linked glycosylation. N-fucosylation, an important type of N-linked glycosylation, consists of α1, 2-, α1, 3/4-, and α1, 6-fucosylation epitopes. They are catalyzed by specific N-fucosyltransferases (FUTs) (Chen et al., 2013). FUT8 is the only key enzyme that catalyzes α1, 6-fucosylation by forming α1, 6-glycosidic bond to transfer the guanosine diphosphate-fucose (GDP-Fuc) to the sixth carbon atom of the innermost N-acetylglucosamine (GlcNAc) of the N-sugar chain core (Miyoshi et al., 1999). Aberrant α1, 6-fucosylation is closely linked to proliferation in colorectal cancer (Muinelo-Romay et al., 2008), ovarian serous adenocarcinoma (Takahashi et al., 2000). The elevated FUT8 level in breast cancer has been found to be associated with lymphatic metastasis (Yue et al., 2016). It's reported that many factors can regulate FUT8 expression, such as microRNA (Bernardi et al., 2013) and selective inhibitor of Src tyrosine kinases (Kaminska et al., 2008), etc. However, whether fentanyl could upregulate the FUT8 and α1, 6-fucosylation level, and further influence stemness and EMT in breast cancer is yet to be elucidated.

In the present study, we found that fentanyl promoted stemness and EMT of MCF-7 and MDA-MB-231 cells. In addition, fentanyl upregulated FUT8 and α1, 6-fucosylation level through activating Wnt/β-catenin signaling pathway. Furthermore, the results *in vivo* showed that fentanyl enhanced FUT8 expression and promoted tumor progression.

## Materials and methods

### Cell culture

The human MCF-7 and MDA-MB-231 breast cancer cells were cultured in DMEM/F12 and L15 medium supplemented with 10% fetal bovine serum (Gibco, USA) and 1% penicillin-streptomycin (TransGen Biotech, China) at 37°C in humidified air containing 5% CO_2_, respectively.

### Drugs and reagents

Fentanyl citrate was obtained from Northeast Pharmaceutical Group (China). The sources of reagents were as follows: Sox2, Oct4, N-cadherin, Vimentin, E-cadherin, β-catenin, GSK-3β, CD44, Cyclin D1, and GAPDH antibody (Proteintech, China); Nanog antibody (Abcam, USA); FUT8, c-Jun and VEGF antibody (Santa Cruz, USA); *Lens culinaris agglutinin* (LCA) Lectin, binding of α1, 6-fucosylation epitope. (Vector Laboratories, USA); p-GSK-3β (Ser^9^) antibody (Elabscience, China); LGK-974 (inhibitor of Wnt ligands) (Selleck, USA).

### Sphere formation assay

Cells were seeded onto ultra low attachment 6-well plate (Corning, USA) at a density of 500 cells per well. The medium was supplemented with 2% B27, 20 ng/ml bFGF, and 20 ng/ml EGF. After culture for 12 days at 37°C in 5% CO_2_, the number of tumor-spheres with a diameter of ≥50 μm was counted under a microscope (Olympus BX51, Japan).

### Colony formation assays

500 cells were seeded onto a 35 mm dish. After culture for 10 days, surviving colonies were counted with crystal violet staining. Triplicate independent experiments were carried out.

### Western blot

The proteins from cells and tumor tissues were extracted. Protein concentration was determined with Coomassie Protein Assay Reagent using bovine serum albumin as a standard. Total protein was separated by a 12% SDS-PAGE gel followed by transferring to a nitrocellulose membrane (NC). The membrane was blocked with 5% non-fat dry milk in TBST for 2 h and incubated overnight with primary antibody or LCA Lectin, and then membrane was incubated with HRP-secondary antibodies or HRP-Streptavidin for 1 h. The HRP conjugates were exposed with the ECL (Amersham, USA) detection system, and data was analyzed by Quantity One software (Bio-Rad, USA).

### Transwell migration assay

Migrated breast cancer cells were determined by transwell migration assay. Briefly, 1 × 10^4^ cells/well were cultured in triplicate in the top chambers of 24-well transwell plates (8.0 mm-pore, Corning, USA) and 10% FBS medium were added into the bottom chambers. After culture for 20 h, the migrated cells on the bottom surface of the top chamber membranes were fixed with methanol, stained with crystal violet, and imaged under a microscope.

### Quantitative PCR

Quantitative PCR (qPCR) was used to measure mRNA level. Total RNAs were extracted using Trizol (Takara, Japan) according to the manufacturer's protocol. RNA was reversely transcribed into cDNA using PrimeScriptTMRT reagent kit (TransGen Biotech, China), and then qPCR was performed using TransStart TipTop Green qPCR SuperMix (TransGen Biotech, China) according to the manufacturer's instructions. The primer sequences were as follows: Nanog: 5′-CCT GTG ATT TGT GGG CCT GA-3′ (F), 5′-CTC TGC AGA AGT GGG TTG TTT G-3′ (R); Sox2: 5′-GTG AGC GCC CTG CAG TAC AA-3′ (F), 5′-GCG AGT AGG ACA TGC TGT AGG TG-3′ (R); Oct4: 5′-GCA GAT CAG CCA CAT CGC CC-3′ (F), 5′-GCC CAG AGT GGT GAC GGA GA-3′ (R); N-cadherin: 5′-AAA GAA CGC CAG GCC AAA C-3′(F), 5′-GGC ATC AGG CTC CAC AGT GT-3′(R); E-cadherin: 5′-CAA CGA CCC AAC CCA AGA A-3′(F), 5′-CCG AAG AAA CAG CAA GAG CA-3′(R); Vimentin: 5′-CGT CTC TGG CAC GTC TTG AC-3′(F), 5′-GCT TGG AAA CAT CCA CAT CGA-3′(R). Quantified data was normalized to GAPDH, and the relative quantity was calculated using the 2-ΔΔCT method. Triplicate independent experiments were carried out.

### Immunofluorescent staining

Immunofluorescent staining was performed after fixing the cells with 4% paraformaldehyde for 20 min. After blocking with 1% goat serum (Beyotime, China) for 2 h, the cells were incubated with primary antibodies overnight at 4°C. After washing with PBS, the cells were incubated with FITC-conjugated goat anti-mouse IgG or FITC-conjugated goat anti-rabbit IgG for 1 h at room temperature. Then slides were incubated with DAPI for 10 min at room temperature. Images were obtained under a fluorescence microscope (Olympus BX83, Japan).

### Lectin fluorescent staining

Lectin fluorescent staining was performed after fixing the cells with 4% paraformaldehyde for 20 min. After blocking with 1% goat serum (Beyotime, China) for 2 h, the cells were incubated with rhodamine-labeled LCA Lectin for 2 h at room temperature. After washing with PBS, the slides were incubated with DAPI for 10 min at room temperature. Images were obtained under a fluorescence microscope (Olympus BX83, Japan).

### siRNA transfection

MCF-7 cells (1 × 10^5^) were trypsinized, and seeded onto 6-well plate. When cells reached 70–80% confluence, short interfering RNA of FUT8 (siFUT8): 5′-GUG GAG UGA UCC UGG AUA UTT-3′ (F), 5′-AUA UCC AGG AUC ACU CCA CTT-3′ (R) (Genepharma, China) was transiently transfected into MCF-7 cells using Lipofectamine™ 2000 Reagent (Invitrogen, Carlsbad, CA, USA) according to the manufacturer's protocol. The cells were collected after 48 h transfection for further experiments.

### Immunohistochemical staining

Visible tumors were removed from the mice, and immunohistochemistry was performed on paraffin-embedded sections. Serial sections (4 μm each) were prepared, deparaffinized in xylene and rehydrated in a graded alcohol. After microwaving for 20 min in citrate buffer to expose the antigen, the slides were washed with PBS and incubated in 3% H_2_O_2_ for 10 min at room temperature to block endogenous peroxidase activity. Non-specific binding was blocked with goat serum at room temperature for 30 min before incubation overnight at 4°C with primary antibody. After extensive washing with PBS, slides were incubated with biotinylated secondary antibody for 15 min at room temperature. The slides were then incubated with a streptavidin-peroxidase complex. The signal was visualized with DAB (3, 3′-diaminobenzidine) kit, and the slides were briefly counterstained with hematoxylin. Images were captured with a microscope.

### Xenograft tumor mouse model

Female nude mice (Balb/c-nu/nu) were obtained from Animal Center (Dalian Medical University). The animals (4–6 weeks) were maintained under sterile conditions during the entire experimental period. MCF-7 cells (5 × 10^6^) suspended in 0.2 ml PBS were injected subcutaneously into the right flank. After 7 days, mice were randomly divided into 2 different groups (*n* = 5/group). Fentanyl (0.02 mg/kg of body weight) was subcutaneously administered for 3 weeks with time interval of 24 h. The mice treated with citrate buffer only served as controls. The tumor dimensions were measured every 3 days using a digital caliper. The tumor volume was calculated according to the formula (Volume = 1/2 Length × Width^2^). At the end of the experiment (the 30th day), the tumor mass was weighed. All animal procedures were approved by the Institutional Review Board of Dalian Medical University.

### Statistical analysis

Data were expressed as mean ± SEM of three independent experiments using GraphPad Prism v5.01 (GraphPad Software, La Jolla, CA, USA). The Student's *t*-test was used to make a statistical comparison between groups. Statistical significance was defined as *P* < 0.05.

## Results

### Fentanyl promotes stemness of breast cancer cells

**Figure 1:**
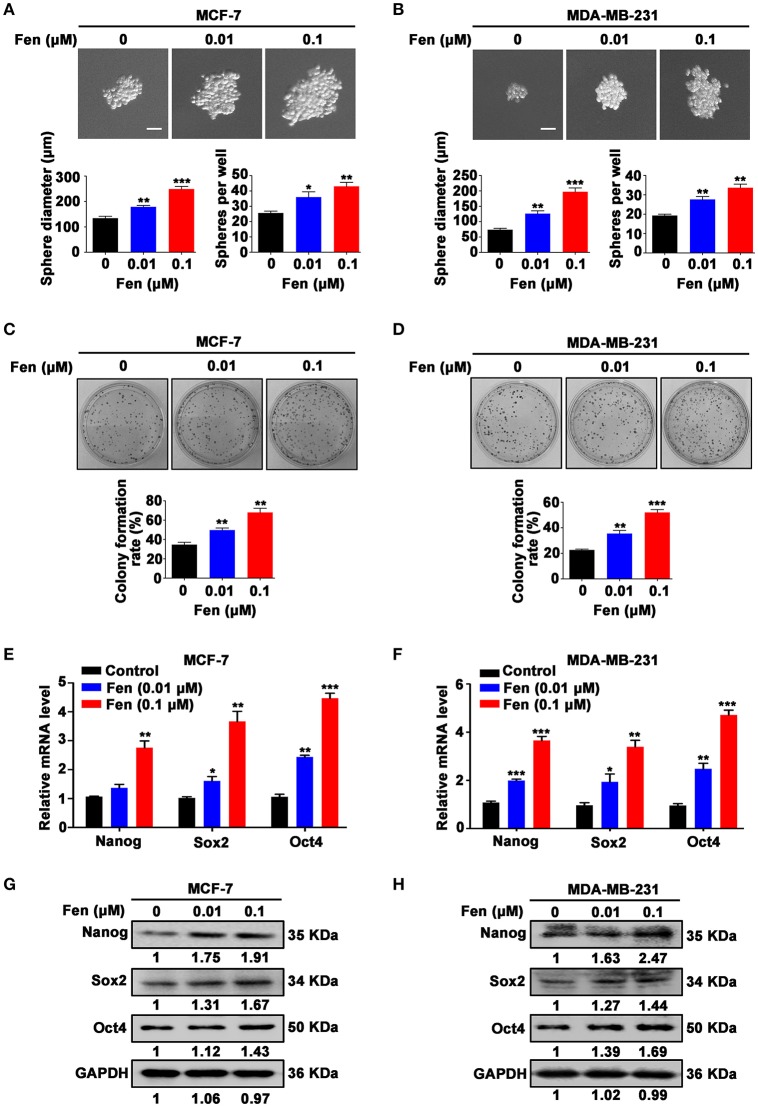
Fentanyl promotes stemness of breast cancer cells. **(A,B)** Representative images of tumor-spheres in MCF-7 and MDA-MB-231 cells under a microscope after treatment with fentanyl (0, 0.01 μM, 0.1 μM) for 12 days. (Scale bars = 50 μm; Magnification, 200×). **(C,D)** Images of colony formation in MCF-7 and MDA-MB-231 cells after treatment with fentanyl for 10 days. **(E,F)** qPCR and (**G,H)** Western blot analysis for Nanog, Sox2, and Oct4 gene and protein expression in MCF-7 and MDA-MB-231 cells treated with fentanyl for 48 h. Data are represented as mean ± SD (*n* = 3). Statistical significant different in sphere diameter, sphere per well and colony formation rate (^*^P < 0.05; ^**^P < 0.01; ^***^P < 0.001).

To analyze the role of fentanyl on stem-like characteristics in breast cancer cells, we performed sphere formation assay in MCF-7 and MDA-MB-231 cells under serum-free and non-adherent conditions with treatment of different concentrations of fentanyl (0, 0.01 μM, 0.1 μM). The results showed that fentanyl induced a significant increase in the size (*P* < 0.01) and number (*P* < 0.05) of tumor-spheres in a dose-dependent manner (Figures 1A,B). Representative images of colony forming assay were shown (Figures 1C,D). There was an obvious stimulating effect of fentanyl on colony formation ability as compared with the untreated cells (*P* < 0.01). We then examined the expression of stemness-related markers. As shown in Figures 1E,F, fentanyl upregulated the expression of Nanog, Sox2 and Oct4 genes by qPCR. In addition, the effect of fentanyl on the protein level was confirmed by Western blot (Figures 1G,H). Significant difference in both mRNA and protein levels normalized to the GAPDH was observed in the control compared to treatment groups (*P* < 0.05). Collectively, these results suggest that fentanyl promotes the stemness of breast cancer cells.

### Fentanyl facilitates EMT of breast cancer cells

**Figure 2:**
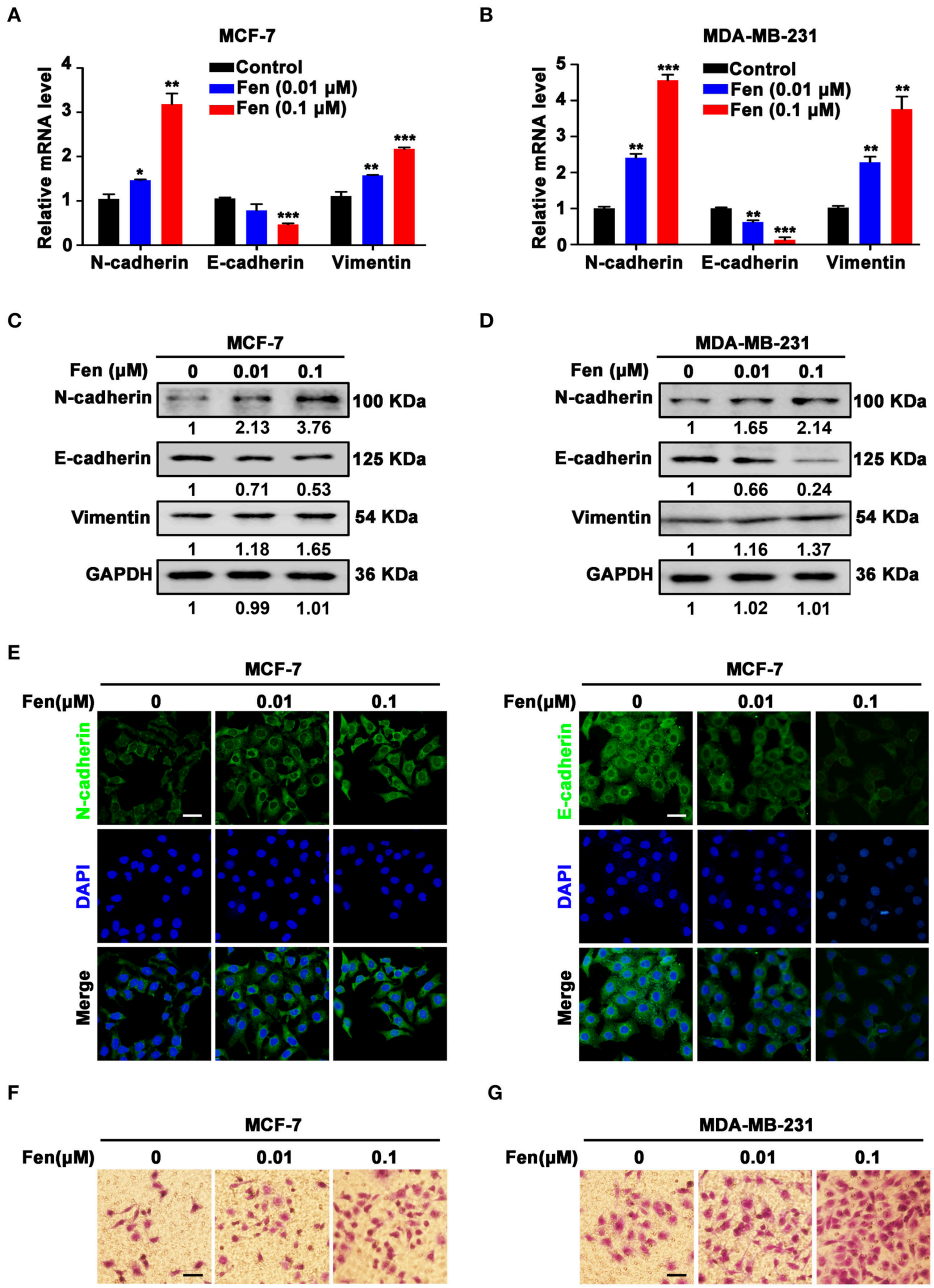
Fentanyl facilitates EMT of breast cancer cells. **(A,B)** qPCR and **(C,D)** Western blot analysis of gene and protein expression of N-cadherin, E-cadherin and Vimentin in MCF-7 and MDA-MB-231 cells treated with fentanyl (0, 0.01 μM, 0.1 μM) for 48 h. **(E)** Representative immunofluorescent images of N-cadherin and E-cadherin of MCF-7 cells treated with fentanyl for 48 h. (Scale bars = 50 μm; Magnification, 200×). **(F,G)** Images of migrated MCF-7 and MDA-MB-231 cells observed under a microscope after fentanyl treatment. (Scale bars = 50 μm; Magnification, 200×). Data are represented as mean ± SD (*n* = 3). Statistical significant different in gene expression (^*^P < 0.05; ^**^P < 0.01; ^***^P < 0.001).

To determine whether fentanyl plays a role in EMT, MCF-7, and MDA-MB-231 cells were treated with different concentrations of fentanyl (0, 0.01 μM, 0.1 μM) for 48 h. The expression of EMT markers N-cadherin, E-cadherin and Vimentin was assessed by qPCR and Western blot. As shown in Figures 2A–D, fentanyl significantly enhanced the expression of N-cadherin (*P* < 0.05) and Vimentin (*P* < 0.01), whereas decreased the expression of E-cadherin (*P* < 0.01). Alterations of N-cadherin and E-cadherin in MCF-7 cells was determined by immunofluorescent staining (Figure 2E). Furthermore, migrated cells were increased in fentanyl treated group compared with the control, confirming the stimulating effect of fentanyl on EMT (Figures 2F,G). These results indicate that fentanyl induces EMT in breast cancer cells.

### Fentanyl upregulates FUT8 expression and α1, 6-fucosylation level in MCF-7 cells

**Figure 3:**
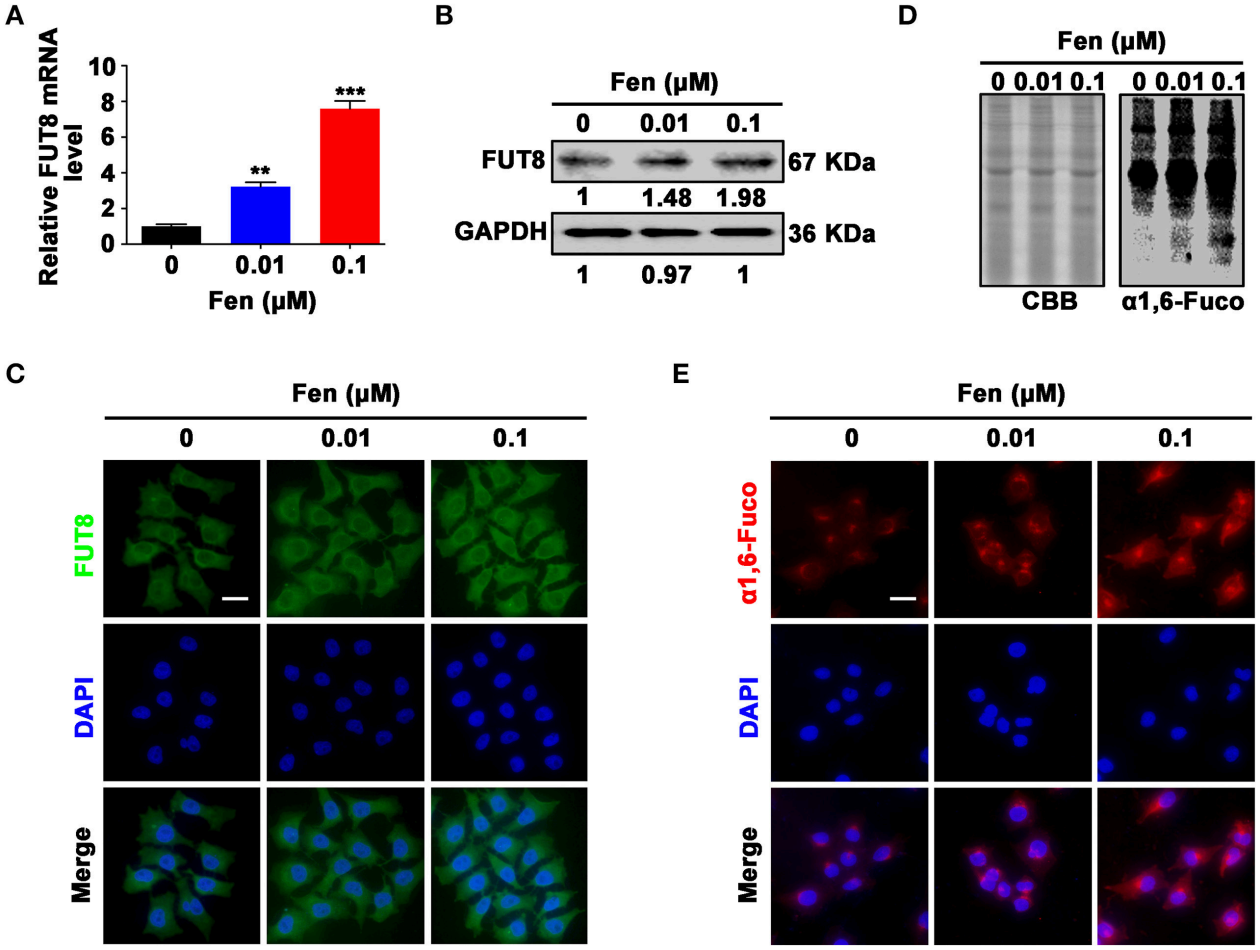
Fentanyl upregulates FUT8 expression and α1, 6-fucosylation level in MCF-7 cells. **(A)** qPCR analysis of FUT8 gene expression. **(B)** Western blot analysis of FUT8 protein expression. **(C)** Representative immunofluorescent images of FUT8 expression. **(D)** Lectin blot analysis of α1, 6-fucosylation level. Coomassie brilliant blue (CBB) was used as a loading control. **(E)** Lectin fluorescent staining of α1, 6-fucosylation expression. (Scale bars = 50 μm; Magnification, 400×). Data are represented as mean ± SD (*n* = 3). Statistical significant different in gene expression (^**^P < 0.01; ^***^P < 0.001).

To explore whether α1, 6-fucosylation catalyzed by FUT8 is involved in EMT and stemness induced by fentanyl, breast cancer cells were treated with different concentrations of fentanyl (0, 0.01 μM, 0.1 μM) for 48 h. The results indicated that fentanyl enhanced FUT8 gene and protein expression compared with the control by qPCR, Western blot and immunofluorescent staining (Figures 3A–C). Furthermore, results showed that fentanyl increased the α1, 6-fucosylation level in a dose-dependent manner by Lectin blot in the treatment groups compared with the control (Figure 3D). The effect of fentanyl on α1, 6-fucosylation level was further confirmed by Lectin fluorescent staining (Figure 3E). In brief, fentanyl promotes FUT8 expression and FUT8-catalyzed α1, 6-fucosylation level in breast cancer cells.

### α1, 6-fucosylation is involved in fentanyl-mediated stemness and EMT

**Figure 4:**
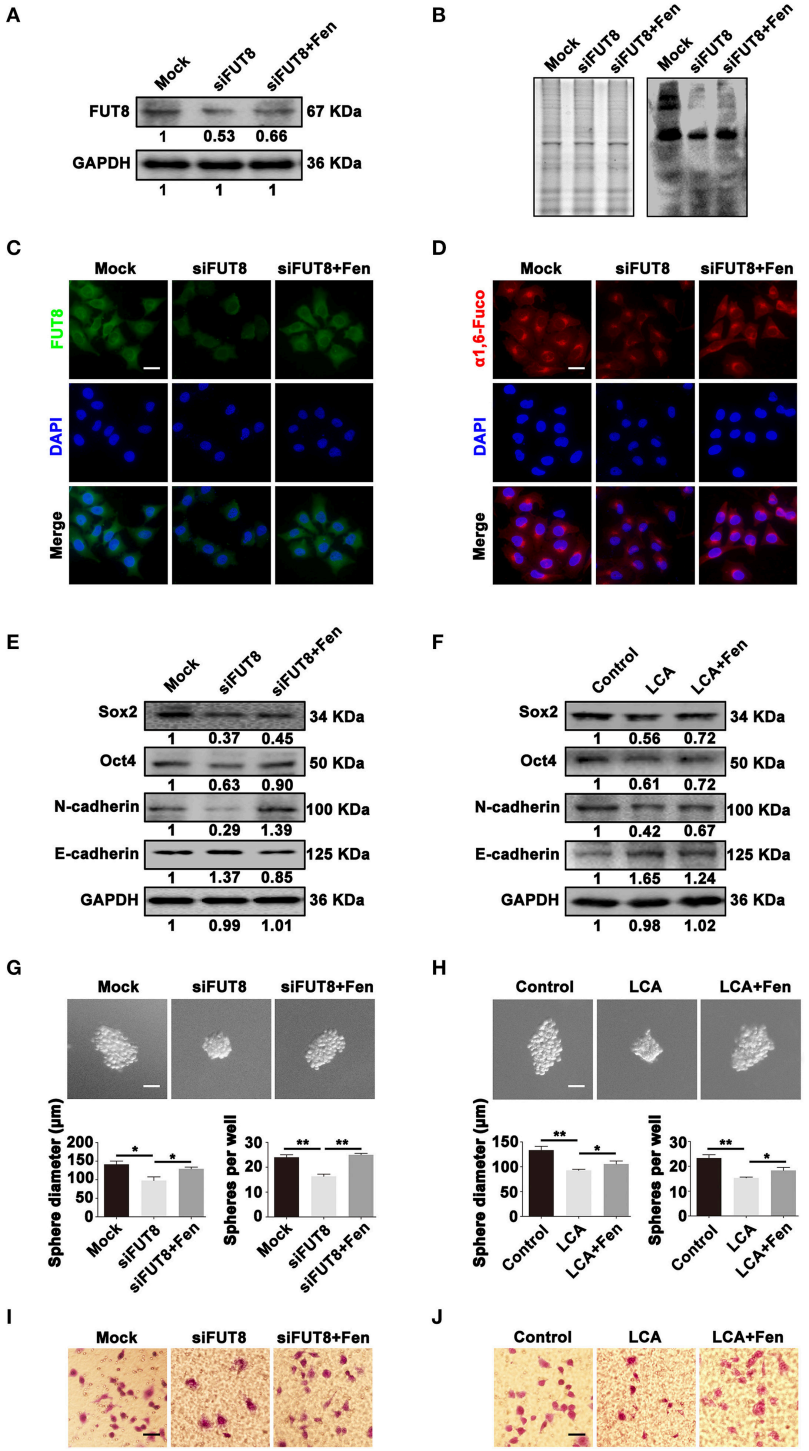
α1, 6-fucosylation is involved in fentanyl-mediated stemness and EMT. **(A,B)** Western blot, Lectin blot **(C,D)** immunofluorescent staining and Lectin fluorescent staining analysis for expression of FUT8 and α1, 6-fucosylation in mock, siFUT8 or siFUT8+fentanyl group. **(E,F)** Western blot analysis for Sox2, Oct4, N-cadherin and E-cadherin potein expression. **(G)** Representative images of tumor-spheres in mock, siFUT8 or siFUT8+fentanyl group observed under a microscope after 12 days of incubation. (Scale bars = 50 μm; Magnification, 200×). **(H)** Images of tumor-spheres in control, LCA or LCA+fentanyl group. **(I)** Images of migrated MCF-7 cells in mock, siFUT8 or siFUT8+fentanyl group observed under a microscope. **(J)** Images of migrated MCF-7 cells in control, LCA and LCA+fentanyl (0.1 μM) groups. (Scale bars = 50 μm; Magnification, 200×). Data are represented as mean ± SD (*n* = 3). Statistical significant different in sphere diameter and sphere per well (^*^P < 0.05; ^**^P < 0.01).

To assess the effects of FUT8 in breast cancer phenotypes, we first knockdown the expression of FUT8 in MCF-7 cells, and the knockdown efficiency was verified by Western blot and immunofluorescent staining (Figures 4A,C). The effect of siFUT8 on the biosynthesis of α1, 6-fucosylation was detected by LCA Lectin blot and Lectin fluorescent staining. The results showed that siFUT8 successfully decreased the level of α1, 6-fucosylation (Figures 4B,D). Furthermore, the results of Western blot showed that decreased or blocked α1, 6-fucosylation by siFUT8 or LCA Lectin treatment, respectively, suppressed the expression of stemness- and EMT-related markers (Figures 4E,F). We then examined whether FUT8 expression and α1, 6-fucosylation level could influence the stemness and EMT of breast cancer cell by sphere formation assay and transwell assay, respectively. As shown in Figures 4G,H, there was a significantly inhibition in size and number of tumor-spheres in siFUT8 transfection (*P* < 0.05) and LCA Lectin blocking groups (*P* < 0.05) compared with the control. Similarly, the migrated cells were also inhibited by siFUT8 transfection and LCA Lectin blocking (Figures 4I,J). Meantime, fentanyl reversed the inhibitory effects of stemness and EMT mentioned above (Figures 4A–J). These findings indicate that FUT8-catalyzed α 1, 6-fucosylation regulates stemness and EMT induced by fentanyl.

### Fentanyl upregulates α1, 6-fucosylation level by activating Wnt/β-catenin signaling pathway

**Figure 5:**
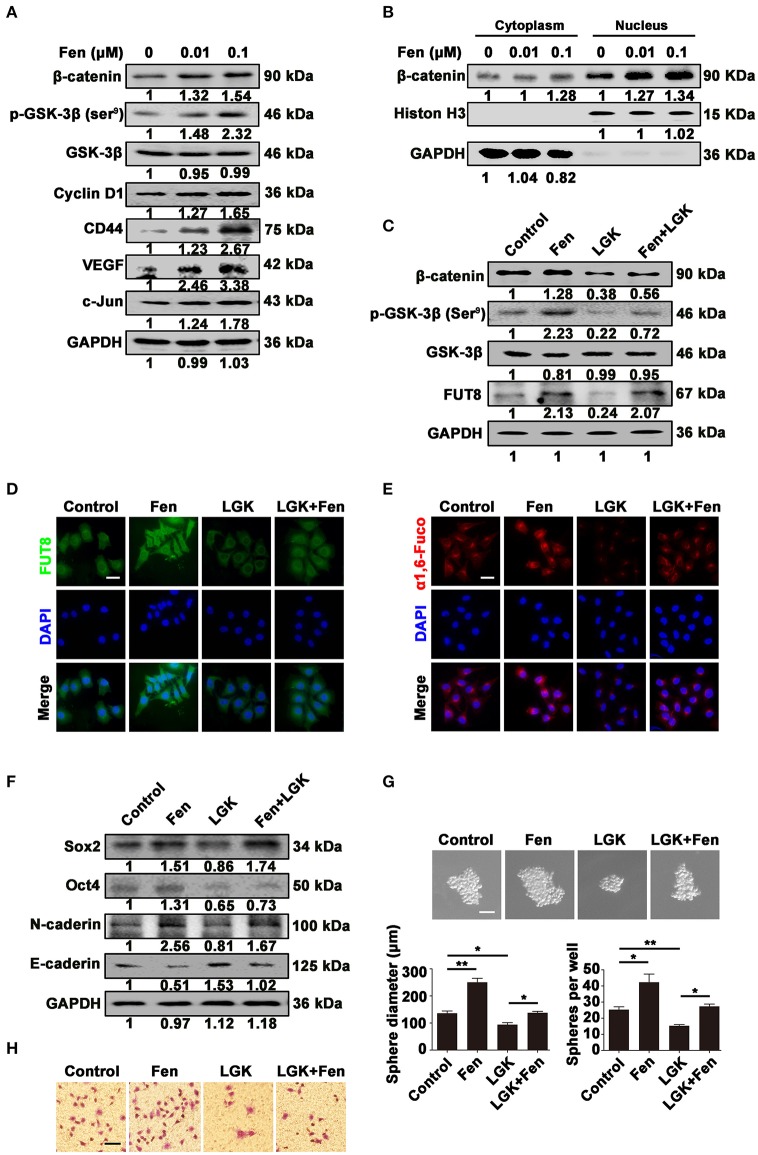
Fentanyl upregulates α1, 6-fucosylation level by activating Wnt/β-catenin signaling pathway. **(A)** Western blot analysis for expression of β-catenin, p-GSK-3β (Ser^9^), GSK-3β, Cyclin D1, CD44, VEGF and c-Jun after treatment with fentanyl (0, 0.01 μM, 0.1 μM) for 48 h. **(B)** Western blot analysis for expression of β-catenin in cytoplasm and nucleus after treatment with fentanyl. GAPDH and Histone H3 were used as cytoplasmic and nuclear control, respectively. **(C)** Western blot analysis for expression of β-catenin, p-GSK-3β (Ser^9^), GSK-3β and FUT8 after treatment with fentanyl, LGK-974 and LGK-974+fentanyl. **(D,E)** Representative immunofluorescent and Lectin fluorescent images of FUT8 and α1, 6-fucosylation. (Scale bars = 50 μm; Magnification, 400×). **(F)** Western blot analysis for Sox2, Oct4, N-cadherin and E-cadherin protein expression. **(G)** Images of MCF-7 cells tumor-spheres, observed under a microscope after treatment with fentanyl, LGK-974 and LGK-974+fentanyl. (Scale bars = 50 μm; Magnification, 200×). **(H)** Images of migrated MCF-7 cells were observed under a microscope. (Scale bars = 50 μm; Magnification, 200×). Data are represented as mean ± SD (*n* = 3). Statistical significant different in gene expression (^*^P < 0.05; ^**^P < 0.01).

We investigated whether Wnt/β-catenin signaling pathway could be activated by fentanyl, followed by detecting the influence of Wnt signaling inhibition on FUT8, α1, 6-fucosylation, stemness and EMT. We found that fentanyl increased the expression of key molecules (p-GSK-3β, β-catenin) and target genes (Cyclin D1, CD44, VEGF, and c-Jun) in Wnt/β-catenin signaling pathway (Figure 5A). In addition, we detected β-catenin expression in the cytoplasm and nucleus separately. Results showed that fentanyl facilitated the translocation of β-catenin from cytoplasm to nucleus indicating the accumulated and activiated form of β-catenin in nuleus (Figure 5B). However, LGK-974 (1 μM), an inhibitor of Wnt ligands, decreased fentanyl-activated β-catenin, p-GSK-3β (Ser^9^) and FUT8 expression (Figure 5C). The inhibitory effect of LGK-974 on FUT8 expression and α1, 6-fucosylation level was further confirmed by immunofluorescent staining and Lectin fluorescent staining (Figures 5D,E). The downregulated expression of Sox2, Oct4, N-cadherin, whereas upregulated expression of E-cadherin induced by LGK-974 was also detected by Western blot (Figure 5F). Furthermore, LGK-974 induced a decrease in the size and number of tumor-spheres, as well as migrated cells (Figures 5G,H). These results suggest that Wnt/β-catenin signaling pathway mediate upregulation of FUT8 and α1, 6-fucosylation upon fentanyl exposure and subsequently promote stemness and EMT in breast cancer cells.

### Fentanyl promotes tumor progression in tumor xenograft

**Figure 6:**
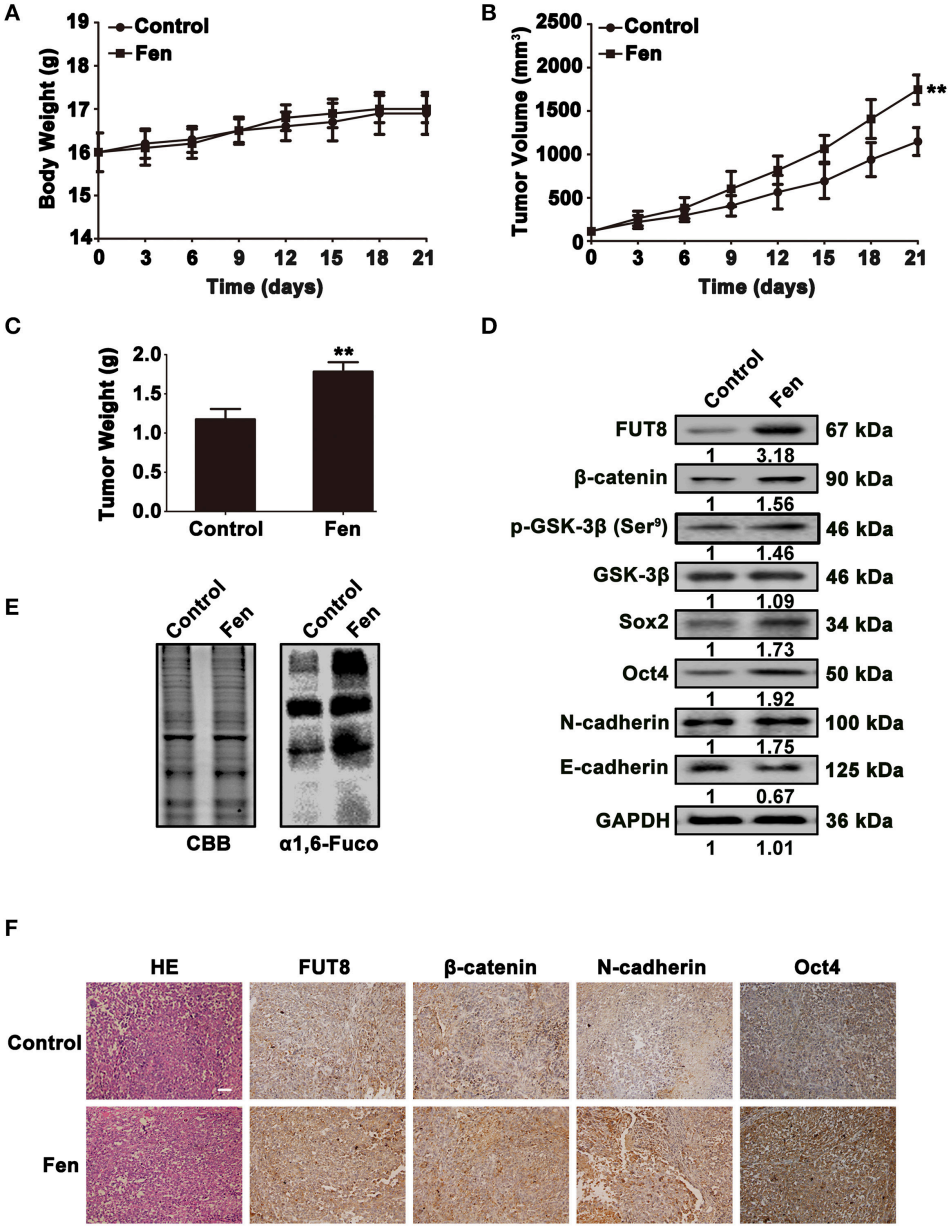
Fentanyl promotes tumor progression in tumor Xenograft. **(A–C)** Histograms of body weight, tumor volume and tumor weight. **(D,E)** Western blot and Lectin blot analysis for FUT8, α1, 6-fucosylation, β-catenin, GSK-3β, p-GSK-3β (Ser^9^), Oct4, Sox2, N-cadherin and E-cadherin expression levels in xenograft tumor tissues. **(F)** H&E and immunohistochemical staining of FUT8, β-catenin, N-cadherin and Oct4. (Scale bars = 100 μm; Magnification, 200×). Data are represented as mean ± SD (*n* = 3). Statistical significant difference (^**^P < 0.01).

To confirm the *in vitro* results, MCF-7 cells were used to establish mouse xenograft tumor models. The tumor volume and weight were analyzed in the control and fentanyl treatment groups. There were no obvious changes in the body weight of the xenograft mice (Figure 6A). However, as shown in Figures 6B,C, fentanyl treatment significantly increased tumor volume and weight in xenografts (*P* < 0.01). The analysis of Western blot showed that fentanyl promoted the expression of FUT8, β-catenin, p-GSK-3β (Ser^9^), Sox2 and Oct4 and N-cadherin, as well as inhibited the expression of E-cadherin (Figure 6D). Lectin blot showed that fentanyl enhanced α1, 6-fucosylation level (Figure 6E). We also detected the expression of FUT8, β-catenin, N-cadherin, and Oct4 in mouse xenograft tumor tissues by immunohistochemistry. As shown in Figure 6F, FUT8, β-catenin, N-cadherin, and Oct4 were highly expressed in fentanyl treatment group compared with the control. These results reveal that fentanyl promotes FUT8 expression, α1, 6-fucosylation level, stemness and EMT of breast cancer cells in tumor xenograft.

## Discussion

Stemness and EMT have been proposed as the driving force of malignant transformation in various cancers, and are closely involved in drug resistant, poor prognosis, etc. (Al-Hajj et al., 2004; Jayachandran et al., 2016). In cancer treatment, chemotherapy and adjuvant analgesics are common therapeutic strategy. However, some chemotherapeutic drugs have been found to promote cancer cell stemness. For example, Zhu et al reported that the 3,3′-diindolylmethane (DIM) activated the Wnt/β-catenin signaling and resulted in gastric cancer cell differentiation and the expression of stemness markers (Zhu et al., 2016). Martins-Neves et al. found that doxorubicin, cisplatin and methotrexate promoted the transition of osteosarcoma cells to stem cell-like phenotypes to cause chemotherapy failure (Martins-Neves et al., 2016). Similarly, some literatures suggested that opioid analgesics contributed to tumor progression. Lennon et al. showed that DAMGO, morphine and fentanyl facilitated EMT in non-small cell lung cancer by activating the opioid receptor (Lennon et al., 2014). Our previous results also demonstrated that morphine induced breast cancer stem cell properties, thus, resistance to doxorubicin and paclitaxel (Niu et al., 2015). Therefore, we hypothesized that fentanyl may have the potential to cause stemness and EMT. Our results showed that fentanyl stimulated tumor-sphere formation in MCF-7 and MDA-MB-231 breast cancer cells. Moreover, the expression of stemness markers were upregulated in both breast cancer cells and mouse xenograft tumor tissues after fentanyl treatment. Additionally, fentanyl facilitated the migration of breast cancer cells. Meanwhile, fentanyl accelerated the EMT *in vitro* and *in vivo* by regulating the expression of the markers. Taken together, our study report, for the first time, that fentanyl promotes stemness and EMT of breast cancer cells. The results of the study provide evidence for the view that opioid analgesics may accelerate tumor progression in the treatment of breast cancer, and further investigation is needed.

Limited evidence showed that glycosylation may sever as a mediator to stemness and EMT. Che et al confirmed that β1, 4-N-acetylgalactosaminyltransferase III (B4GALNT3) regulated GalNAcβ1-4GlcNAc (LacdiNAc) on the epidermal growth factor receptor (EGFR) to promote the stemness and invasiveness of colorectal cancer cells (Che et al., 2014). Our previous study showed that the downregulation of fucosyltransferase IV (FUT4) expression inhibited the biosynthesis of Lewis Y antigen (LeY), which inactivated EGFR and downstream signaling pathways to ultimately decrease EMT in lung cancer cells (Tian et al., 2016). FUT8 knockdown significantly inhibited malignant behaviors including *in vitro* invasion and cell proliferation in lung cancer cells, as well as *in vivo* metastasis and tumor growth (Chen et al., 2013). In the present study, we found that the increased expression of FUT8 by fentanyl promoted stemness and EMT in breast cancer cells; whereas downregulated FUT8 and α1, 6-fucosylation level by siFUT8 transfection inhibited stemness and EMT. Meanwhile, these inhibitory effects were confirmed by blocking α1, 6-fucosylation on the cellular surface by LCA Lectin incubation. Based on these findings, we speculate that FUT8 and α 1, 6-fucosylation may serve as potential targets for breast cancer treatment.

Aberrant activation of Wnt/β-catenin signaling pathway is associated with a variety of pathological alterations, including cancer, metabolic disorder and neurodegenerative diseases (Libro et al., 2016; Ma and Hottiger, 2016). Some studies have demonstrated that Wnt/β-catenin signaling pathway was involved in the properties of CSCs, and was closely correlated with EMT (Reya and Clevers, 2005; Ghahhari and Babashah, 2015; Zhang et al., 2015). For example, Lettini et al. proved that heat shock protein TRAP1 regulated stem-like signature of colorectal cancer cells by Wnt/β-catenin signaling pathway through the modulation of the expression of Wnt ligands and the control of β-catenin ubiquitination/phosphorylation (Lettini et al., 2016). It is also demonstrated that gamma-tubulin complex protein-5 (GPC5) competitively bound Wnt3a to inactivate the Wnt/β-catenin signaling pathway and inhibited the EMT of lung cancer cells (Wang S. et al., 2016). Our results showed that fentanyl increased the key molecules and the target genes of the Wnt/β-catenin signaling pathway. In addition, the expression of FUT8, α1, 6-fucosylation, Sox2, Oct4, and N-cadherin was significantly reduced by treatment with Wnt/β-catenin signaling inhibitor LGK-974. These data suggest that Wnt/β-catenin signaling pathway mediated the upregulated FUT8 and α1, 6-fucosylation level, which contributed to the promotion of breast cancer stemness and EMT.

In conclusion, this study suggested fentanyl upregulated FUT8 and α1, 6-fucosylation level through activation of Wnt/β-catenin signaling pathway, thereby, induce stemness and EMT of breast cancer cells. Our study results illustrate a new role of fentanyl in promoting stemness and EMT when used to relief pain, which will guide the application of fentanyl in breast cancer treatment.

## Author contributions

HY, MY, HJ, and JY conducted the cell and animal studies. ZL and IY wrote the paper. QY and QW designed the project and modified the paper.

### Conflict of interest statement

The authors declare that the research was conducted in the absence of any commercial or financial relationships that could be construed as a potential conflict of interest.

## Abbreviations

CSCs: cancer stem-like cells
EMT: epithelial-mesenchymal transition
FUT8: fucosyltransferase VIII
LCA: Lens Culinaris Agglutinin.
